# Efficacy and safety of onabotulinumtoxinA in patients with overactive bladder: subgroup analyses by sex and by serum prostate-specific antigen levels in men from a randomized controlled trial

**DOI:** 10.1007/s11255-021-02962-z

**Published:** 2021-07-22

**Authors:** Osamu Yokoyama, Masashi Honda, Tomonori Yamanishi, Yuki Sekiguchi, Kenji Fujii, Kyoko Kinoshita, Takashi Nakayama, Akikazu Ueno, Takao Mogi

**Affiliations:** 1 Department of Urology, Faculty of Medical Science, University of Fukui, Fukui, Japan; 2 Department of Urology, Tottori University Faculty of Medicine, Tottori, Japan; 3 Department of Urology, Continence Center, Dokkyo Medical University, Tochigi, Japan; 4 Yokohama Motomachi Women’s Clinic LUNA, Kanagawa, Japan; 5 Specialty Medical and Development, GlaxoSmithKline, Tokyo, Japan; 6 Biostatistics, GlaxoSmithKline, Tokyo, Japan

**Keywords:** OnabotulinumtoxinA, Overactive bladder, Randomized controlled trial, Men, Prostate-specific antigen

## Abstract

**Purpose:**

We aimed to assess onabotulinumtoxinA treatment outcomes by sex in patients with overactive bladder (OAB) and then explore the impact of serum prostate-specific antigen (PSA) levels in men.

**Methods:**

Patients inadequately managed with OAB medications were randomized to receive single-dose onabotulinumtoxinA (100 U) or placebo intravesical injection in a phase III trial in Japan. We performed subgroup analyses by sex and post-hoc subgroup analyses using male PSA categories.

**Results:**

In women (*n* = 186), onabotulinumtoxinA demonstrated statistically significant and clinically relevant improvements in all urinary symptoms at Week 12. In men with lower PSA (< 1.5 ng/mL, *n* = 40), onabotulinumtoxinA also showed numerically greater reductions in urinary symptom frequency than placebo; the between-group differences (onabotulinumtoxinA minus placebo) in change from baseline in the average daily number at Week 12 for urinary incontinence (UI), urgency UI, micturition, urgency, and nocturia were − 1.43, − 1.79, − 2.81, − 2.45, and − 0.32 episodes, respectively. In men with higher PSA (≥ 1.5 ng/mL, *n* = 22), onabotulinumtoxinA did not reduce urinary symptom frequency. Some patients treated with onabotulinumtoxinA showed elevated post-void residual urine volume at Week 2 (≥ 200 mL): 4 of 91 women, none of the men with lower PSA and 3 of 11 men with higher PSA.

**Conclusions:**

OnabotulinumtoxinA was efficacious and well tolerated in women and in men with lower PSA levels. Given our post-hoc subgroup analyses which suggested that onabotulinumtoxinA treatment is a good treatment option for OAB males with lower PSA levels, future studies having prostate volume data with larger sample size are warranted to verify our findings.

**ClinicalTrials.gov Identifier:**

NCT02820844 (first posted July 1, 2016). https://clinicaltrials.gov/ct2/show/NCT02820844.

## Introduction

Overactive bladder (OAB) is a symptom syndrome with urinary urgency, usually accompanied by frequency and nocturia, with or without urgency urinary incontinence (UUI) [1]. The initial therapies based on the international OAB guidelines [2–4] are behavioral therapy followed by pharmacologic therapy (oral antimuscarinics and oral β_3_-adrenoceptor agonists).

Intradetrusor injection of onabotulinumtoxinA 100 U (BOTOX^®^, Allergan, an AbbVie Company, North Chicago, Illinois, USA) is one of the next treatment options for patients who failed primary therapies.Although several studies have previously reported the efficacy and safety of onabotulinumtoxinA in OAB patients, most of these studies enrolled mostly women. As shown in a recent review [5], few studies have focused on the outcomes of onabotulinumtoxinA treatment exclusively in men [6–9], and none of those that did were controlled studies. These studies assessed the impact of prostate enlargement as a potential confounding factor for onabotulinumtoxinA treatment outcomes, because the urological pathophysiology of OAB is differs between women and men. However, the factors that may impact the degree of effectiveness of onabotulinumtoxinA in male patients remain unclear.

Regarding antimuscarinic treatments, several studies have suggested that men with OAB who have lower serum prostate-specific antigen (PSA) levels and smaller prostates may benefit for their OAB symptoms [10–12]. Thus, we hypothesized that PSA levels in men with OAB might have an impact on onabotulinumtoxinA treatment outcomes similar to antimuscarinic treatment. Furthermore, a high level of placebo effect in patients with OAB has been reported [13], and it would be more valuable to assess the difference from placebo on OAB efficacy. We conducted subgroup analyses using phase III trial data [14] to assess the onabotulinumtoxinA treatment outcomes by sex and then explored the impact of PSA levels in men. These are the first subgroup analyses by sex, and in men by PSA level, in patients with OAB based on a randomized controlled trial (RCT) with placebo for onabotulinumtoxinA.

## Materials and methods

### Subjects and study design

A full description of the study design has been published elsewhere [14]. This randomized controlled phase III trial was conducted from 2016 to 2018 at 53 sites in Japan (ClinicalTrials.gov NCT02820844), in compliance with Good Clinical Practice regulations. This study consisted of two phases: the double-blind treatment phase (randomized, placebo-controlled design) and the open-label treatment phase. The study included patients who were inadequately managed with OAB medications. Patients aged ≥ 20 years who had ≥ 3 episodes of UUI per day, ≥ 8 micturitions per day, and a post-void residual (PVR) urine volume of < 100 mL at baseline, without using clean intermittent catheterization or indwelling catheterization, were eligible for the study. To exclude prostate cancer patients, the PSA levels of male patient at baseline were measured in a central laboratory. Men with significantly higher PSA levels (> 10 ng/mL) were excluded, but those with PSA levels of > 4 and ≤ 10 ng/mL were included unless they were clinically determined by the investigator (expert urologists) to have prostate cancer. Patients with coexisting benign prostatic hyperplasia (BPH) were allowed to participate, but those with urethral obstruction and/or bladder outlet obstruction (BOO) were excluded, again as clinically judged by the investigator with no definite criteria such as maximum urinary flow rate (*Q*_max_).

The enrolled patients were randomized on a 1:1 basis to receive a double-blind treatment with onabotulinumtoxinA 100 U or placebo injection across 20 sites in the detrusor muscle. For the post-hoc analyses (GlaxoSmithKline Data Reuse Request number 101996), we used the single-dose double-blind data up to 12 weeks as the patients were allowed to receive the open-label treatment with onabotulinumtoxinA 100 U after the double-blind phase.

Efficacy outcomes for the post-hoc analyses included bladder diary variables and patient-reported outcomes (PROs). PROs were assessed using the Overactive Bladder Symptom Score (OABSS) [15] and King’s Health Questionnaire (KHQ) [16]. Adverse events (AEs) and PVR urine volumes were evaluated to assess safety.

### Statistical analyses

The aim of the subgroup analyses was to assess heterogeneity in the treatment effects between the subgroups. Following pre-specified subgroup analyses by sex for urinary incontinence (UI) and volume voided per micturition, we performed post-hoc subgroup analyses. In men, the PSA categories defined for statistical analysis were as follows: men with lower PSA levels (< 1.5 ng/mL) and men with higher PSA levels (≥ 1.5 ng/mL). Based on the evidence from several studies [11, 17–20], we selected 1.5 ng/mL as the cut-off value for PSA levels with the aim of identifying men with an enlarged prostate (> 30 mL) [21].

All efficacy analyses were performed using a full analysis set (all randomized patients who had at least one post-baseline efficacy assessment, FAS). The primary endpoint for this trial was the change from baseline in the average daily number of UI episodes at Week 12. The mean changes in urinary symptoms from baseline to Week 12 were estimated using a mixed model for repeated measures (MMRM). The mean changes in PROs from baseline to Week 12 were analyzed using analysis of covariance (ANCOVA). We calculated the 95% confidence interval to estimate the between-group differences for each subgroup for all efficacy variables, and *p* values were not provided because these post-hoc subgroup analyses were exploratory manner.

Safety analyses were conducted among patients who received a single dose of the study drug (safety population). The summary statistics of the safety variables were provided.

All analyses were conducted using SAS version 9.4 (SAS Institute, Cary, North Carolina, USA). Multiplicity was not considered in the post-hoc exploratory analyses.

## Results

### Baseline characteristics

A total of 250 patients were randomized; of these, 248 were included in the FAS population. In this study, the number of women (*n* = 186) was higher than that of men (*n* = 62). Of the 62 male patients, 40 had lower PSA levels and 22 had higher PSA levels. The median PSA level (range) showed no major differences between the onabotulinumtoxinA group (1.10 [0.21, 9.78] ng/mL) and the placebo group (1.26 [0.01, 4.28] ng/mL). However, the mean PSA level (standard deviation [SD]) was higher in the onabotulinumtoxinA group (2.35 [2.60] ng/mL) than in the placebo group (1.37 [1.00] ng/mL).

The baseline demographics and disease characteristics were similar across the subgroups and treatment groups with the exception of the following trends in men with higher PSA levels (Table 1), the patients in the onabotulinumtoxinA group were associated with older age, and the patients in the placebo group were associated with less frequent UI and UUI episodes, and lower KHQ role limitations score than those in the other groups. Overall, the baseline characteristics of each group were generally comparable.

**Table 1:** Baseline demographics and safety results

	Analyses by sex				Analyses by PSA categories			
	Women		Men		Men with lower PSA levels		Men with higher PSA levels	
	Placebo	OnabotA 100 U	Placebo	OnabotA 100 U	Placebo	OnabotA 100 U	Placebo	OnabotA 100 U
Baseline demographics	(*n* = 94)	(*n* = 92)	(*n* = 30)	(*n* = 32)	(*n* = 19)	(*n* = 21)	(*n* = 11)	(*n* = 11)
Age (years)	66.0 ± 12.09	64.1 ± 12.63	66.9 ± 12.67	70.2 ± 10.80	67.7 ± 13.85	68.4 ± 12.06	65.6 ± 10.84	73.5 ± 7.20
Weight (kg)	56.11 ± 10.90	56.33 ± 10.61	68.87 ± 12.11	63.84 ± 9.36	70.30 ± 13.22	64.57 ± 9.89	66.39 ± 10.01	62.45 ± 8.52
Height (cm)	153.76 ± 5.83	153.29 ± 6.38	164.93 ± 5.48	163.79 ± 7.80	165.69 ± 6.16	163.87 ± 8.34	163.62 ± 3.96	163.65 ± 7.02
OAB history (years)	4.51 ± 4.26	4.91 ± 4.10	3.18 ± 3.10	6.09 ± 6.54	3.68 ± 3.61	6.67 ± 7.61	2.30 ± 1.76	4.99 ± 3.83
PVR urine volume < 100 mL, *n* (%)	94 (100)	92 (100)	30 (100)	32 (100)	19 (100)	21 (100)	11 (100)	11 (100)
Number of daily episodes								
UI	6.09 ± 3.60	7.18 ± 4.11	6.19 ± 4.66	6.50 ± 6.39	6.89 ± 5.05	6.38 ± 5.34	5.00 ± 3.86	6.73 ± 8.34
UUI	5.77 ± 3.48	6.65 ± 4.00	5.50 ± 3.75	6.29 ± 6.44	5.89 ± 3.72	6.11 ± 5.39	4.85 ± 3.94	6.64 ± 8.39
Micturition	12.16 ± 2.95	11.93 ± 3.09	14.46 ± 3.89	13.00 ± 5.08	14.07 ± 4.01	12.65 ± 4.32	15.18 ± 3.72	13.67 ± 6.48
Urgency	8.70 ± 3.52	8.82 ± 4.17	12.17 ± 4.98	10.23 ± 6.18	12.39 ± 4.33	9.83 ± 5.00	11.82 ± 6.17	11.00 ± 8.21
Nocturia	1.72 ± 1.38	1.51 ± 1.48	2.30 ± 1.42	2.27 ± 1.33	2.56 ± 1.51	2.38 ± 1.39	1.88 ± 1.24	2.12 ± 1.27
Volume voided per micturition (mL)	135.96 ± 49.74	134.25 ± 49.63	113.35 ± 42.90	125.77 ± 54.47	113.30 ± 44.16	127.11 ± 50.64	114.25 ± 40.77	123.20 ± 63.70
KHQ domain scores								
Role limitations	60.28 ± 26.45	63.59 ± 28.81	62.64 ± 30.75	60.42 ± 30.16	72.22 ± 29.70	60.32 ± 30.49	46.97 ± 26.69	60.61 ± 30.98
Social limitations	45.86 ± 29.98	46.68 ± 31.05	48.85 ± 23.26	46.53 ± 29.39	50.93 ± 23.20	43.92 ± 30.93	45.45 ± 24.07	51.52 ± 26.89
OABSS total score	11.5 ± 2.23	11.3 ± 1.88	12.1 ± 1.66	12.2 ± 1.80	12.5 ± 1.47	12.0 ± 1.92	11.5 ± 1.81	12.5 ± 1.57
AEs over 12 weeks, *n* (%)	(*n* = 94)	(*n* = 92)	(*n* = 30)	(*n* = 32)	(*n* = 19)	(*n* = 21)	(*n* = 11)	(*n* = 11)
Any AE	50 (53)	58 (63)	14 (47)	18 (56)	8 (42)	10 (48)	6 (55)	8 (73)
Urinary tract infection*	7 (7)	14 (15)	2 (7)	2 (6)	1 (5)	0	1 (9)	2 (18)
Nasopharyngitis	8 (9)	13 (14)	3 (10)	2 (6)	2 (11)	1 (5)	1 (9)	1 (9)
Dysuria	3 (3)	10 (11)	0	2 (6)	0	0	0	2 (18)
Urinary retention**	1 (1)	3 (3)	1 (3)	4 (13)***	0	0	1 (9)	4 (36)***
Residual urine volume increased	0	4 (4)	0	3 (9)	0	1 (5)	0	2 (18)
Cystitis	2 (2)	4 (4)	0	0	0	0	0	0
Hematuria	3 (3)	2 (2)	1 (3)	1 (3)	0	0	1 (9)	1 (9)
PVR urine volume category at Week 2, *n* (%)	(*n* = 94)	(*n* = 91)	(*n* = 30)	(*n* = 32)	(*n* = 19)	(*n* = 21)	(*n* = 11)	(*n* = 11)
< 100 mL	90 (96)	71 (78)	30 (100)	22 (69)	19 (100)	17 (81)	11 (100)	5 (45)
≥ 100 mL to < 200 mL	4 (4)	16 (18)	0	7 (22)	0	4 (19)	0	3 (27)
≥ 200 mL to < 350 mL	0	4 (4)	0	2 (6)	0	0	0	2 (18)
≥ 350 mL	0	0	0	1 (3)	0	0	0	1 (9)

All data are expressed as mean ± standard deviation except otherwise indicated. Common adverse events with 3% or greater incidence in any treatment group of overall population

*AE* adverse event, *KHQ* King’s Health Questionnaire, *OAB* overactive bladder, *OABSS* Overactive Bladder Symptom Score, *OnabotA* onabotulinumtoxinA, *PSA* prostate-specific antigen, *PVR* post-void residual, *UI* urinary incontinence, *UUI* urgency urinary incontinence

*Positive urine culture with bacteriuria count greater than 10^5^ cfu/mL and leukocyturia greater than 5 per high power field regardless of symptoms

**PVR 350 mL or greater regardless of symptoms, or between 200 and less than 350 mL with associated symptoms requiring clean intermittent catheterization in the investigator opinion

***Of 4 patients, 1 patient was reported to have a “feeling of residual urine” by the investigator. This verbatim term was coded as “urinary retention” in preferred term of MedDRA although this patient's PVR was less than 200 mL

### Efficacy analysis

**Fig. 1:**
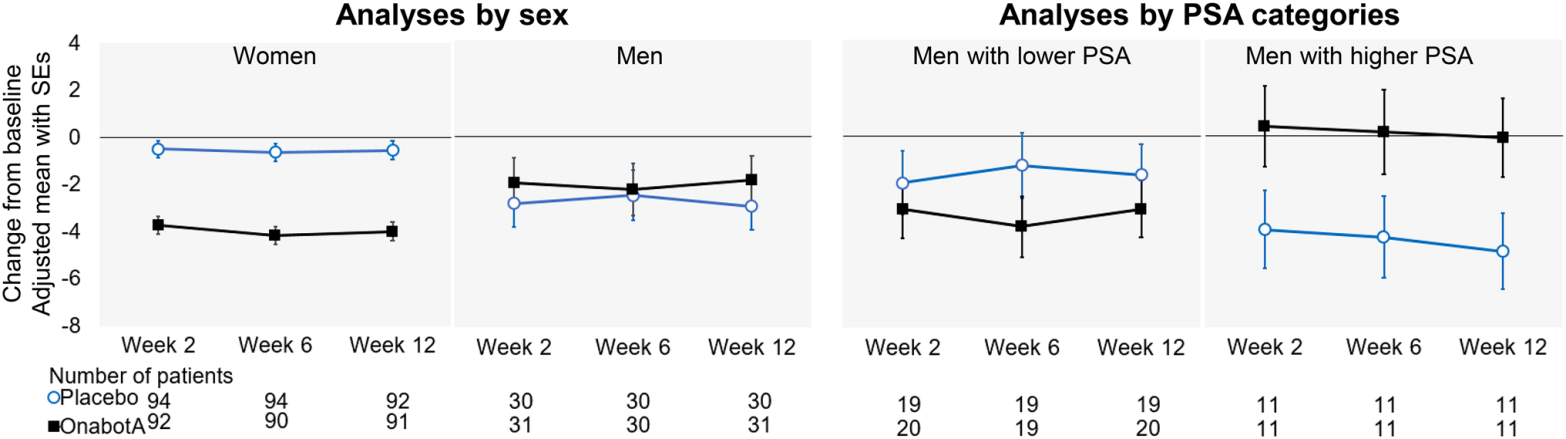
Plots for the change from baseline up to Week 12 in the average daily number of urinary incontinence (UI) episodes. Values: adjusted mean; error bars: standard error. Data by sex were analyzed using a mixed model for repeated measures (MMRM) with treatment, site, visit, treatment-by-visit interaction, baseline value, and baseline-by-visit interaction as fixed effects. The data by prostate-specific antigen (PSA) categories were analyzed using an MMRM with PSA category, treatment-by-PSA category interaction, and treatment-by-PSA category-by-visit interaction as fixed effects in addition to the above fixed effects. When it is assumed that the treatment difference for UI were − 1.79 or − 1.43 and 3.5 for its standard deviation (SD), post-hoc power with 39 participants using two-sample *t*-test will be 34% and 24% power at the two-sided significance level of 5% to detect treatment differences in men with lower PSA. Here, − 1.79 and 3.5 are the effect size and SD assumed in the study protocol for the overall population, respectively, and − 1.43 is the observed treatment difference adjusted mean in men with lower PSA levels

Figure 1 shows the plots for the primary endpoint for individual treatment groups up to Week 12. OnabotulinumtoxinA showed greater decreases than placebo in women but not in men. When men were divided into two subgroups according to PSA levels, onabotulinumtoxinA showed numerically greater decreases than in the placebo group in men with lower PSA levels. By contrast, in men with higher PSA levels, onabotulinumtoxinA showed no notable changes from baseline, whereas placebo showed reductions. Because this trend was also noted in measures other than UI episode, the following results on between-group difference should not be interpreted that onabotulinumtoxinA worsened efficacy outcomes in men with higher PSA levels.

**Fig. 2:**
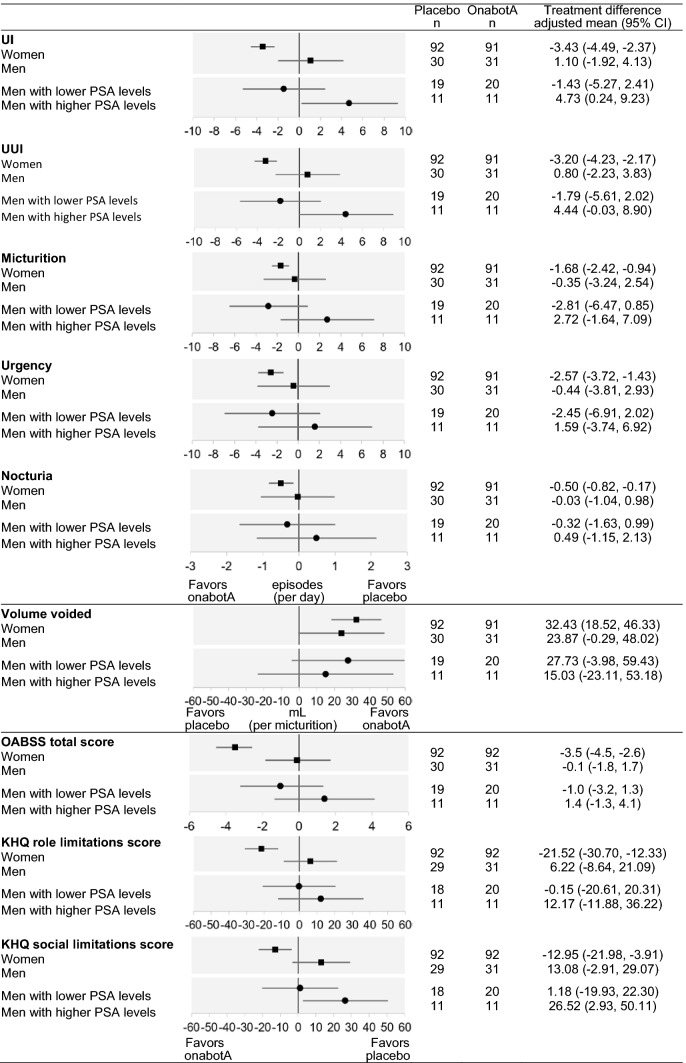
Forest plots for the between-group differences in the change from baseline at Week 12 for urinary symptoms and patient-reported outcomes. Closed square: analyses by sex, closed circle: analyses by prostate-specific antigen (PSA) categories. Values: adjusted mean; error bars: 95% confidence interval. The data for urinary incontinence (UI) and urgency urinary incontinence (UUI) by sex were analyzed using a mixed model for repeated measures (MMRM) with treatment, site, visit, treatment-by-visit interaction, baseline value, and baseline-by-visit interaction as fixed effects, whereas other data for urinary symptoms by sex were analyzed using an MMRM with the baseline frequency of UUI episodes (≤ 9 or ≥ 10 per 3 days) in addition to the above fixed effects. The data for the urinary symptoms by PSA categories were analyzed using an MMRM with above the fixed effects by adding the PSA category, treatment-by-PSA category interaction and treatment-by-PSA category-by-visit interaction as fixed effects. The data for the patient-reported outcomes by sex were analyzed using analysis of covariance (ANCOVA) with terms for treatment, site, baseline value, and baseline frequency of UUI episodes (≤ 9 or ≥ 10 per 3 days) as fixed effects. The data for the patient-reported outcomes by PSA categories were analyzed using ANCOVA with terms for treatment, site, baseline value, the baseline frequency of UUI episodes (≤ 9 or ≥ 10 per 3 days), PSA category and treatment-by-PSA category interaction as fixed effects

Figure 2 shows the forest plots for the between-group differences in the change from baseline in all efficacy outcomes: urinary symptoms and PROs at Week 12. Assessment by sex and by PSA levels in Fig. 2 showed a trend similar to that in Fig. 1. OnabotulinumtoxinA demonstrated clinically relevant improvements in all efficacy outcomes in women. However, there was no notable difference between the onabotulinumtoxinA and placebo groups in almost all efficacy outcomes in men. After dividing men according to PSA levels, onabotulinumtoxinA consistently showed greater improvements than the placebo group for all urinary symptoms in men with lower PSA levels, although these were numeric improvements. With regard to the PROs, no clear trend toward improvement was shown in the KHQ subscale scores; however the OABSS total score, a PRO measure reflecting overall OAB symptoms in a single result, did tend to improve in men with lower PSA levels.

By contrast, onabotulinumtoxinA did not result in greater improvements than the placebo among men with higher PSA levels. For UI, UUI, micturition, urgency, nocturia episodes, OABSS total score and KHQ scores, favorable outcomes were observed in the placebo group compared with the onabotulinumtoxinA group in men with higher PSA levels. As an exception, onabotulinumtoxinA consistently improved the volume voided per micturition in all subgroups.

### Safety analysis

OnabotulinumtoxinA was well tolerated in women as well as in the overall population (Table 1) [14]. The proportion of patients with a PVR urine volume ≥ 200 mL (at Week 2) and incidence of urinary retention (over the 12 weeks) after receiving onabotulinumtoxinA was higher in men than in women. However, the incidence of urinary tract infection (over the 12 weeks) was higher in women than in men in the onabotulinumtoxinA group. The subgroup analysis by PSA levels showed that all of those men had higher PSA levels: of the eleven men with higher PSA levels treated with onabotulinumtoxinA, three had a PVR urine volume of ≥ 200 mL (two of three men had urinary tract infection) and four had an AE of urinary retention.

## Discussion

This study provides the first subgroup analysis by sex based on an RCT with placebo and subsequently focused on the impact of PSA levels on onabotulinumtoxinA treatment outcomes in men with OAB. The results demonstrated that onabotulinumtoxinA treatment was effective and well tolerated in men with lower PSA levels. Notably, onabotulinumtoxinA consistently improved all analyzed urinary symptoms and main PROs in men with lower PSA levels, although the improvements were lower than those seen in women. In men with higher PSA levels, however, onabotulinumtoxinA was less effective than the placebo for almost all endpoints and was associated with an increased frequency of elevated PVR urine volumes. Our post-hoc subgroup analysis suggests that onabotulinumtoxinA is a good treatment option for men with lower PSA levels as for women. We believe that these results in Japanese patients are globally applicable because onabotulinumtoxinA acts locally and is thus unlikely to be susceptible to ethnic differences.

Why did onabotulinumtoxinA show lower efficacy and safety in men with higher PSA levels in comparison with men with lower PSA levels? To begin with, men with higher PSA levels might have some level of enlarged prostate that causes the frequency, urgency and incontinence by simply putting pressure on the bladder. It is well known that prostate volume is the most important factor contributing to PSA elevation in men without clinically detectable prostate cancer, even though there are other numerous confounding factors including prostatic inflammation and prostatic calculi [22]. Furthermore, previous studies have indicated that detrusor overactivity isinduced by increased sensory input from the prostatic urethra in patients with prostate enlargement [23, 24]. Our results in men with higher PSA levels are similar to those that were previously published in a post-hoc subgroup analyses based on RCT data of antimuscarinics which showed that tolterodine extended-release was efficacious against OAB symptoms in men with lower PSA levels and small prostates [11, 12]. Men with higher PSA levels, who are considered to have a large prostate volume, may have a complicated pathophysiologic mechanism that could worsen OAB symptoms in men. Our study also suggested that the risk of urinary retention was lower in men with lower PSA levels than in men with higher PSA levels. Although the possibility that the elevated PVR was a result of the detrusor underactivity caused by the onabotulinumtoxinA injection alone cannot be ruled out, the degree of BOO at baseline, which is often associated with prostate enlargement, may also be a contributing factor to urinary retention. Our post-hoc subgroup analysis may support the recommendation that treatment of prostate enlargement/BOO conditions should be prioritized before onabotulinumtoxinA treatment is initiated because of the potential to confound the degree of treatment response as well as for potential safety concerns. These factors may have led to the lower efficacy and safety in the onabotulinumtoxinA group among men with higher PSA levels in our results, although more information is needed (urodynamics, bladder wall thickness, history of urinary tract infections, and cystoscopy outcomes). We hope that our results can trigger more meaningful discussions on the clinical and etiological diversity of OAB.

An important limitation of our analyses is that the direct impact of enlarged prostates was not assessed, although several studies have shown that PSA is an appropriate indicator of prostate enlargement [21, 25, 26] and have reported a correlation between PSA levels and prostate volumes. Our recommendation for future clinical trials for OAB is to obtain data in men not only on PSA levels but also on prostate size as the exclusion criteria. The PLESS study also reported that baseline PSA and/or prostate volume are useful tools to aid physicians and decision makers in predicting outcomes and choosing therapy, especially for BPH patients [27]. Furthermore, if it is possible to obtain urodynamic data including bladder contractility and BOO indices in RCTs, these data will provide further insights into the treatment outcomes of OAB therapies in men. Another limitation is that our analyses were conducted on a post-hoc basis. The potential dangers of over-interpretation of unplanned subgroup analyses were well known. In addition, our subgroup analyses are based on relatively small sample sizes, especially in men with higher PSA levels; and finally, patients with BOO were excluded based on clinical judgment by the investigator with no definite criteria. Hence, caution should be exercised when interpreting the results of our exploratory post-hoc subgroup analyses and larger RCTs are needed to verify the findings of this study in the future.

## Conclusions

OnabotulinumtoxinA was efficacious and well tolerated in women and in men with lower PSA levels. Given our post-hoc subgroup analyses which suggested that onabotulinumtoxinA treatment is a good treatment option for OAB males with lower PSA levels, future studies having prostate volume data with larger sample size are warranted to verify our findings.

## Data Availability

Anonymized individual participant data and study documents can be requested for further research from www.clinicalstudydatarequest.com.
